# Ferroptosis-related gene AKR1C1 predicts the prognosis of non-small cell lung cancer

**DOI:** 10.1186/s12935-021-02267-2

**Published:** 2021-10-26

**Authors:** Fangfang Huang, Yushi Zheng, Xiaoling Li, Hui Luo, Lianxiang Luo

**Affiliations:** 1 Guangdong Medical University, Zhanjiang, 524023 Guangdong China; 2 The First Clinical College, Guangdong Medical University, Zhanjiang, 524023 Guangdong China; 3 Experimental Animal Center, Guangdong Medical University, Zhanjiang, 524023 Guangdong China; 4 The Marine Biomedical Research Institute, Guangdong Medical University, Zhanjiang, 524023 Guangdong China; 5 The Marine Biomedical Research Institute of Guangdong Zhanjiang, Zhanjiang, 524023 Guangdong China; 6 Southern Marine Science and Engineering Guangdong Laboratory (Zhanjiang), Zhanjiang, 524023 Guangdong China

**Keywords:** Non-small cell lung cancer, Ferroptosis, AKR1C1, Bioinformatics analysis, Biomarker

## Abstract

**Background:**

Ferroptosis is a newly discovered mode of cell death distinct from apoptosis and necrosis, and its activation contributes to anticancer therapy in a variety of cancers. However, the prognostic value of ferroptosis-related genes in non-small cell lung cancer (NSCLC) remains to be further investigated.

**Methods:**

NSCLC transcriptome mRNA-seq data set and corresponding clinical data set were downloaded from the Cancer Genome Atlas (TCGA). Then, bioinformatics approaches were subsequently employed to identify potential prognostic markers. Finally, the effects of candidate markers on NSCLC cell proliferation, migration, and ferroptosis were assessed by CCK8, colony formation, wound-healing assay, and functional assays related to ferroptosis.

**Results:**

A total of 37 common differentially expressed genes were screened based TCGA database. Six overall survival associated genes (ENPP2, ULK1, CP, LURAP1L, HIC1, AKR1C1) were selected to build survival model, of which hub gene AKR1C1 was with high expression and low ferroptosis level in NSCLC tumor. Further research showed that AKR1C1 was related with many pathways involved in the process of ferroptosis and associated with diverse cancer-infiltrating immune cells. Moreover, the results of in vitro experiments indicated that the expression of AKR1C1 was upregulated in NSCLC cell lines, and silencing AKR1C1 can inhibit the proliferation and migration of NSCLC cells and promote the occurrence of ferroptosis.

**Conclusions:**

Our study revealed the potential role of ferroptosis-related gene AKR1C1 in NSCLC, which can be used for prognostic prediction in NSCLC.

**Supplementary Information:**

The online version contains supplementary material available at 10.1186/s12935-021-02267-2.

## Introduction

Lung cancer is one of the most common types of cancer in the world, accounting for approximately 14.3% and 8.4% of the total number of cancer cases in men and women, respectively, and is the leading cause of cancer death worldwide (18.0% of the total number of cancer deaths) [1, 2]. NSCLC is a major form of lung cancer, accounting for approximately 85% of all lung cancer cases [3, 4]. In recent years, significant progress has been made in targeted therapy for NSCLC, but the poor survival rate of patients with lung cancer has not improved [5]. Therefore, more accurate biomarkers are urgently needed to develop new therapeutic strategies.

Recently, gene expression analysis by microarray technology has shown a great potential space in cancer research and been widely applied to molecular diagnosis, cancer classification, new drug targets discovery and tumor response prediction [6, 7]. It has been recognized as a promising diagnostic and prognostic tool [6, 7]. Through microarray analysis, many studies have confirmed differentially expressed genes (DEGs) in various types of cancer, thereby determining their unacknowledged roles in biological processes, molecular functions, and different pathways [8–10]. For example, Tang et al. utilize miRNA microarray technology to reveal that miR-208a can affect the proliferation and radiosensitivity of NSCLC cells by targeting p21, which may be a potential therapeutic target for NSCLC patients [10]. Therefore, some key genes and pathways in NSCLC can be identified by microarray technology.

As a newly discovered mode of cell death different from apoptosis and necrosis, ferroptosis was triggered by iron-dependent peroxidation accumulation and first used to describe the form of cell death induced by small molecule erastin [11]. It is mainly characterized by cell volume contraction, increased mitochondrial membrane density, and no typical apoptotic and necrotic manifestations [11]. A variety of genes have been found to be involved in the regulation of ferroptosis [12, 13]. For example, studies have reported that glutathione peroxidase 4 (GPX4) is a key regulator of ferroptosis, which acts as a unique intracellular antioxidant enzyme that exerts phospholipid peroxidase activity and directly reduces peroxidized phospholipids produced in cell membranes, and inactivation of GPX4 can lead to the accumulation of peroxides as well as the occurrence of ferroptosis [11, 12, 14]. In recent years, accumulating evidence has shown that activation of ferroptosis contributes to anticancer therapy in various types of cancer [15–17]. For example, erastin has been found to enhance the effect of cisplatin in NSCLC, providing a new strategy for the treatment of drug-resistant tumors [18]. Therefore, investigating the ferroptosis-related gene expression profile and its prognostic value in NSCLC may develop new strategies for the treatment of NSCLC.

In this study, the transcriptome dataset and corresponding clinical dataset from the Cancer Genome Atlas (TCGA) were merged with the ferroptosis-related genes for systematically bioinformatics analysis. The aim of the study was to identify differentially expressed genes (DEGs) from these datasets in order to identify potential biomarkers by constructing protein‑protein interaction (PPI) networks, and to verify and investigate potential biomarkers in vitro.

## Materials and methods

### Data collection and preprocess

NSCLC transcriptome mRNA-seq data set and corresponding clinical data set (including 999 tumor samples and 103 normal samples) were downloaded from TCGA (https://cancergenome.nih.gov). Then we obtained 291 ferroptosis-related genes from the human gene database (Gene Cards) with the keywords “Ferroptosis” (https://www.genecards.org/) [19] and FerrDb database (http://www.zhounan.org/ferrdb) [20]. After filtering out low expression genes of the mRNA matrix, we merged 269 ferroptosis related genes with the mRNA expression matrix from TCGA. Subsequently, R packages “edgeR” [21] and “DESeq2” [22] were applied to differential gene expression analysis. In order to improve the accuracy of our analysis, only genes identified by both analysis with an adjusted *P*-value > 0.05 and |log2 fold change (FC)| ≥ 2 was considered as differentially expressed and defined as DEGs.

### Functional enrichment analysis of DEGs

In order to further understand the biological functions and significantly enriched metabolic pathways of the DEGs, we performed Gene Ontology (GO) and the Kyoto Encyclopedia of Genes and Genomes (KEGG) [23] enrichment analysis. GO analysis classified the DEGs into three categories, including biological process (BP), cellular component (CC), and molecular function (MF). In this study, GO terms and KEGG pathways with an adjusted *P*-value < 0.05 were considered significantly enriched in DEGs. GO analysis and KEGG analysis were visually display through R software (version 4.0.3).

Gene Set Enrichment Analysis (GSEA) is a computational method that determines whether an a priori defined set of genes shows statistically significant, concordant differences between two biological states via GSEA software version 4.0.3 [24]. We utilized it to analyze the function and potential pathway of signature genes. GSEA was used to further validate the functional enrichment of signature genes. The false discovery rate (FDR) < 25% and nominal *P* < 0.05 were regarded as the cut-off criteria. We set the cut-off criterion to a false discovery rate (FDR) < 25% and nominal *P* < 0.05.

### Protein–protein interaction network construction and module analysis

The STRING database was the online database resource search tool for the retrieval of interacting genes/proteins, which collected and reassessing available experimental data on protein–protein interactions [25]. We used STRING database to construct a protein–protein interaction (PPI) network for the 37 DEGs associated with ferroptosis. Then we imported results into Cytoscape software (version 3.8.2) and run a Cytoscape plugin, CytoHubba application, to select the hub genes [26].

### Survival analysis

We sorted out complete 697 cases of clinical information from 999 patients with NSCLC tumor. Univariate Cox was performed to select the ferroptosis-related genes whose parameter *P*-values less than 0.05 for subsequent analysis. The further selection of clinical prognosis-associated candidate genes was implemented with the R package “rbsurv” [27]. Next, the six selected genes were used to construct a risk model by multiple stepwise Cox regression analysis to predict prognosis in NSCLC tumor patients. The risk score of hub genes was established as Risk score = (exprgene1 × coefficientgene1) + (exprgene2 × coefficientgene2) + ⋯ + (exprgene6 × coefficientgene6). To validate the gene risk signature in the internal validation data sets, we calculated the risk score for each patient in the complete TCGA cohort. Then according to the corresponding median risk score, we divided the NSCLC patients of TCGA cohorts into two groups (high and low risk). Kaplan–Meier analysis was applied to calculate the overall survival (OS) difference between two groups. For the survival analysis of each gene, R package “Survival” was used to conduct survival analysis and the R package “survminer” determined the optimal cut-off expression value and generated the Kaplan–Meier plots. Additionally, multivariate Cox analysis was presented to testify whether the prognosis power of the risk assessment model was independent of other clinical characteristics. Time-dependent ROC curve was used to analyze to evaluate the predictive power of the gene signature and prediction accuracy of this Cox risk model. The receiver operating characteristic curve (ROC) was constructed by predicting the probability of a diagnosis being of high or low integrated score of significant hub gene expression. The area under curve (AUC) analysis was used to evaluate the predictive power of the gene signature and prediction accuracy of this Cox risk model.

### Signature gene validation and analysis

#### Oncomine database analysis

We analyzed the signature gene AKR1C1 expression level in various types of cancer, especially in lung cancer, on the Oncomine database (https://www.oncomine.org/). Oncomine database is an online cancer database with powerful analytical capabilities for computing gene expression signatures, clusters and gene-set modules, automatically extracting biological insights from the data [28]. The mRNA expression difference between tumors and normal tissues were analyzed with thresholds as follows: *P*-value of 0.05, fold change of 2, gene ranking of Top 10 % and the data from mRNA.

#### The ferroptosis potential index (FPI) model

The ferroptosis potential index (FPI) is a model evaluating the ferroptosis level and revealing the functional roles of ferroptosis. In most tumors, high FPI values were often associated with clinical features, and cancer metastasis, recurrence, outcome, and drug sensitivity [29]. Utilizing the model, we evaluated the FPI value of AKR1C1 in NSCLC tumor, revealing the its ferroptosis level AKR1C1 in NSCLC.

#### Kaplan-Meier plotter database analysis

We verified the prognostic value of AKR1C1 again in Kaplan-Meier plotter database (http://kmplot.com/analysis/). Kaplan‐Meier plotter database is an online analysis tool containing microarray profiles and mRNA‐seq data with patients’ survival information, including OS and RFS [30]. The clinical relevance of AKR1C1 mRNA expression in NSCLC cancer patients was analyzed by Kaplan-Meier survival plots. The hazard ratio (HR) with 95 % confidence interval and log-rank *P*-values were calculated.

### Immune Infiltrates and ferroptosis

We further evaluated the infiltrating scores of 5 immune cells and the activities of 4 immune-related pathways with the single-sample gene set enrichment analysis (ssGSEA) in the R package “GSEA” [31]. Additionally, the correlations between AKR1C1 expression and the abundance of immune infiltrates were explored by the Gene module in the TIMER database (https://cistrome.shinyapps.io/timer/). TIMER database is a comprehensive tool established for systematically analyzing the abundance of tumor-infiltrating immune cells (TIICs) from gene expression profiles across diverse types of cancer [32]. We next quantified the number of 22 immune cells and the expression levels of AKR1C1 in human lung tumor tissue. The analysis was display on R software (version 4.0.1). We used two-sided Fisher’s exact test and *P*<0.05 was considered significant.

### Immunohistochemical (IHC) staining

Paraffin-embedded sections were incubated at 60 °C for 1 h and subsequently deparaffinized with xylene and hydrated with graded ethanol. The slides were then boiled in citrate buffer for antigen retrieval, and 3% H_2_O_2_ was used to block the activity of endogenous peroxidase. They were subsequently blocked with 5 % goat serum for 0.5 h. Sections were incubated with rabbit anti-human AKR1C1 antibody (1:100; ABclonal, Wuhan, China) overnight at 4 °C, followed by incubation with HRP-conjugated secondary antibody for 0.5 h at 37 °C. The detection of immunohistochemistry was performed using the DAB substrate kit (Sangon, Shanghai, China), and the nucleus were counterstained with hematoxylin. IHC staining scores were evaluated using image J software. The Score has four grades, 4 = high positive, 3 = positive, 2 = low positive and 1 = negative.

### RNA extraction and reverse transcription–quantitative polymerase chain reaction (RT–qPCR)

Total RNA was extracted from cells using TRIzol® reagent (Invitrogen, Thermo Fisher Scientific, Inc., USA) according to the manufacturer’s protocol. One microgram of total RNA was reversed to cDNA using transcriptor first strand cDNA synthesis kit (Roche, Shanghai, China), followed by SYBR-Green real-time PCR (Roche, Shanghai, China). RT-PCR reactions were performed according to the manufacturer’s instructions. The 2 ^−ΔΔCt^ method was used to evaluate the mRNA expression. Relative expression was calculated and normalized to GAPDH. The sequences of oligonucleotide primers were synthesized by Sangon (Shanghai, China) and the forward and reverse primer sequences were as follows: GAPDH forward, 5′-ATCATCCCTGCCTCTACTGG-3′ and reverse, 5′-GTCAGGTCCACCACTGACAC-3′; AKR1C1 forward, 5′-CATGCCTGTCCTGGGATTT-3′ and reverse, 5′-AGAATCAATATGGCGGAAGC-3′.

### Cell culture and transfection

Human NSCLC cell lines (A549, PC-9, H1975) and human normal bronchial epithelial cells (BEAS-2B) were purchased from American Type Culture Collection (ATCC). All cells were maintained in RPMI-1640 medium (Gibco, GrandIsland, USA) containing 10 % fetal bovine serum (FBS; Gibco, GrandIsland, USA) and 1% penicillin-streptomycin (Gibco, GrandIsland, USA) and were incubated under 37 °C and 5 % CO_2_ conditions.

AKR1C1-siRNA was obtained from Sangon Biotech (Shanghai, China) for silencing the expression of AKR1C1. In this study, the AKR1C1-siRNA sequence was as follows: 5′-AAGCTTTAGAGGCCACCAAAT-3′. Cells were inoculated in 12-well plates at a density of 8 × 10^4^ cells/well until 60%–70% cell confluence for transfection. And cells were transfected with AKR1C1-siRNA/NC-siRNA using siRNA Transfection Reagent (Polyplus, France) to a final concentration of 5 nM. Finally, target protein expression level was analyzed in cells transfected for 48 h. Successfully transfected cells were used for subsequent experiments.

### Cell proliferation assay

Cell Counting Kit-8 (CCK8; Beyotime Biotechnology, Shanghai, China) was used to perform cell proliferation analysis. Cells were inoculated in 96-well plates at a density of 4 × 10^3^ cells/well and cultured for 0, 24, 48, and 72 h. CCK-8 solution was added and incubated in incubator for 1.5 h. Then the absorbance value was measured at 450 nm to calculate the number of viable cells.

### Colony formation assay

Colony formation assay was used to perform cell proliferation analysis. Cells were inoculated in 12-well plates at a density of 2000 cells/well and incubated under 37 °C and 5% CO_2_ conditions for 1 week. One week later, the cells were washed with phosphate-buffered saline (PBS), fixed in 1 mL/well 4% paraformaldehyde (Leagene Biotechnology, Beijing, China) for 20 min, and stained with 1% crystal violet staining solution (Solarbio, Beijing, China) for 10 min at room temperature. Finally, the crystal violet staining solution was slowly washed off with running water and dried in air.

### Wound-healing assay

A typical wound-healing assay was performed to assess the migration ability of A549 and H1975 cells. Cells were inoculated in 12-well plates at a density of 1 × 10^5^ until the cells were completely confluent, and the confluent monolayer was subsequently damaged with a yellow sterile pipette tip. Cells were washed three times with PBS to remove detached cells, and then cultured in serum-free medium for 24 h. Images were collected at 0 and 24 h. Experiments were repeated at least three times.

### Iron assay

FerroOrange (1 µmol/L, Dojindo, Japan) was added to transfected A549 and H1975 cells and then incubated under 37 °C and 5% CO_2_ conditions for 30 min. Finally, cells were observed under a fluorescence microscope (BioTek Cytation 5, BioTek, USA).

### Lipid peroxidation assay

C11-BODIPY 581/591 (10 µM; ABclonal, Wuhan, China) was added to transfected A549 and H1975 cells and incubated under 37 °C and 5% CO_2_ conditions for 1 h. At the end of the incubation, the cells were washed twice with PBS and digested with trypsin, then the cells were resuspended in PBS containing 5% FBS and finally analyzed by flow cytometry.

### Western Blot assay

Proteins were extracted from cells, and cell lysates were prepared with RIPA lysate (Solarbio, Beijing, China) added with PMSF, followed by protein quantification using the BCA protein assay kit (Sangon Biotech, Shanghai, China). Proteins were subsequently separated with 10 % SDS-PAGE and transferred to nitrocellulose membranes. 5 % bovine serum albumin (BSA) was used to block the membranes and then the membranes were incubated with primary antibodies overnight at 4 °C. The next day, after the membranes were washed three times with TBST, horseradish peroxidase-labeled secondary antibodies (1:4000) were added for 1 h at room temperature, after which they were washed three times with TBST. Finally, color development was performed using BeyoECL Moon (Beyotime Biotechnology, Shanghai, China).

### Statistical analysis

Data are expressed as mean ± standard deviation (SD). Statistical analysis was performed by using GraphPad Prism analysis software. The t-test was used to assess the difference between the two groups and a value of *P* < 0.05 indicates a statistically significant difference, * indicates *P* < 0.05; ** indicates *P* < 0.01; *** indicates *P* < 0.001.

## Results

### Identification of common differentially expressed genes (DEGs)

**Fig. 1:**
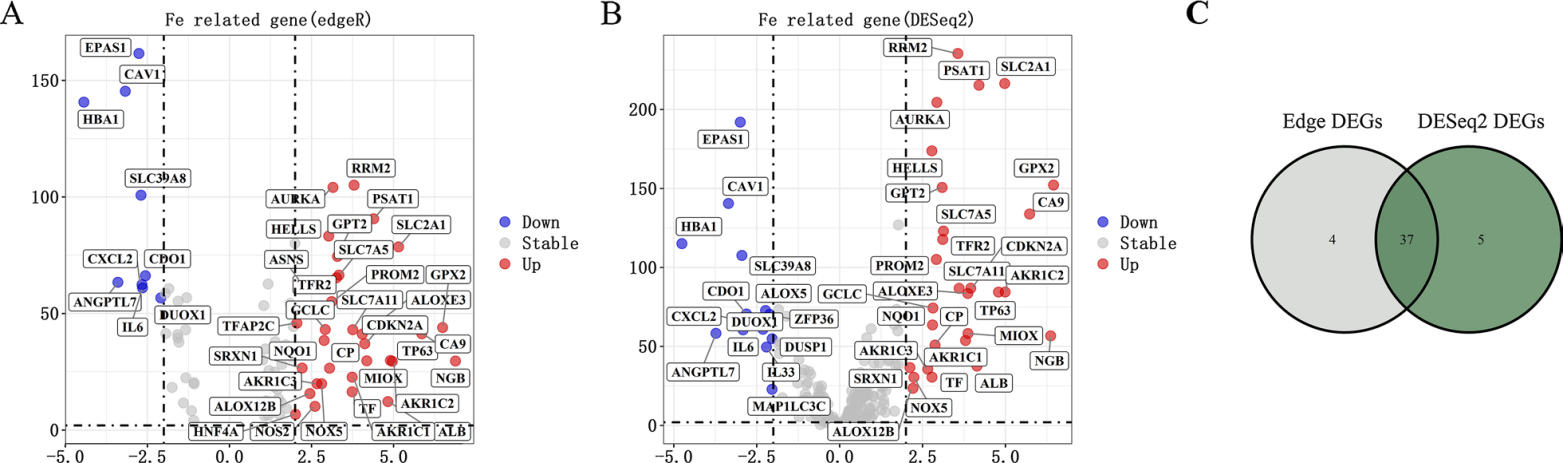
Verification of common differential genes in NSCLC. **A**, **B** The volcano plots visualize the DEGs by R package (**A**) “Edger” (**B**) and DeSeq2. |log2FC| > 2 and *P* < 0.05. The red nodes represent upregulated genes while the blue nodes represent downregulated genes. **C** Common DEGs

Sorting out the data of TCGA, we carried out differential expression analysis on the NSCLC gene expression matrix. Utilizing R packages “edgeR” filtering (log2FC > 2 and FDR < 0.05), we screened out 41 DEGs, including 32 up-regulated and 9 down-regulated gene (Fig. 1A). In the same way, we screened out 42 DEGs by R package “DESeq2”, including 28 up-regulated and 14 down-regulated gene (Fig. 1B). Subsequently, these DEGs were subjected to Venn diagram analysis, 37 common DEGs in the intersection of both analysis results were identified and selected for further analysis (Fig. 1C). In order to make the results more intuitive, we visualized them on R software.

### Analysis of biological properties and pathways related to the DEGs

**Fig. 2:**
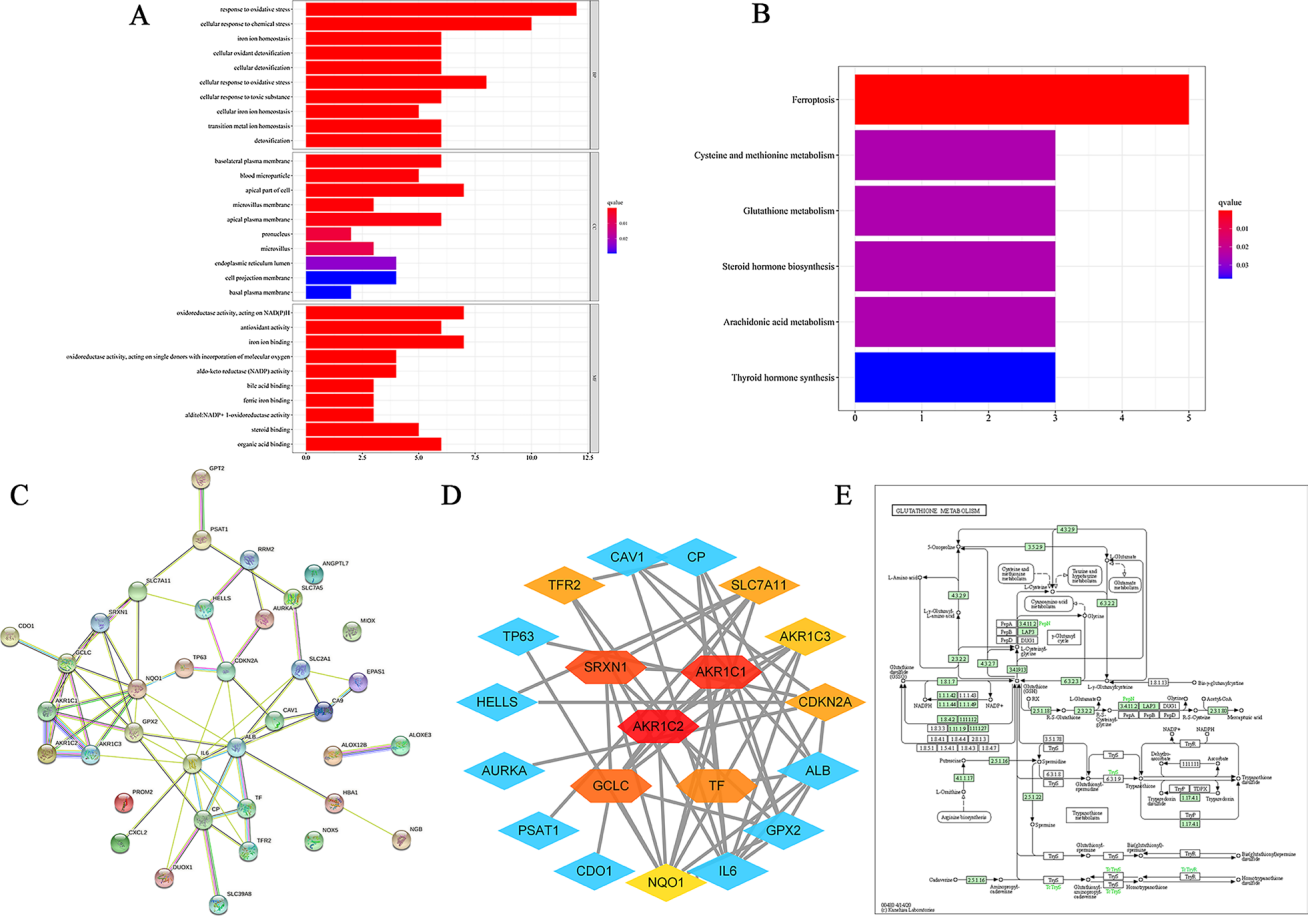
Enrichment analysis and PPI network construction of different expressed ferroptosis-related genes. **A** Bar plot of GO enrichment in cellular component terms, biological process terms, and molecular function terms. **B** Bar plot of KEGG enriched terms. **C** A protein–protein interaction network was constructed from the STRING database online. **D** The hub genes were screened with cytoHubba on Cytoscape. **E** GSH pathway in which the GSE is involved

Moreover, we conducted GO analysis and KEGG pathway enrichment analysis of DEGs from differential analysis to explore their potential biological functions and pathways in NSCLC. The results of GO analysis in Fig. 2A showed that DEGs were significantly related to response to oxidative stress, cellular response to chemical stress, iron ion homeostasis, cellular oxidant detoxification, cellular detoxification, cellular response to oxidative stress, cellular response to toxic substance, cellular iron ion homeostasis, transition metal ion homeostasis, detoxification, basolateral plasma membrane, blood microparticle, apical part of cell, microvillus, membrane apical plasma membrane, pronucleus, microvillus, endoplasmic reticulum lumen, cell projection membrane, basal plasma membrane, oxidoreductase activity, acting on NAD(P)H, antioxidant activity, iron ion binding, oxidoreductase activity, acting on single donors with incorporation of molecular oxygen, aldo-keto reductase (NADP) activity, bile acid binding, ferrie iron binding, alditol: NADP + 1-oxidoreductase activity, steroid binding-organic acid binding. These biological oxidation functions are closely related to the main molecular function of these ferroptosis regulation. The results of KEGG analysis in Fig. 2B suggested that DEGs were significantly associated with ferroptosis, cysteine and methionine metabolism, glutathione metabolism, steroid hormone biosynthesis, arachidonic acid metabolism, thyroid hormone synthesis. The ferroptosis pathway and glutathione metabolism pathway are important pathways regulating ferroptosis, so we displayed glutathione metabolism pathway in Fig. 2E based on KEGG analysis results. According to above analysis results, we confirmed that the 34 selected genes are related to ferroptosis and suggested the role of glutathione biosynthesis and metabolism in NSCLC.

### Regulatory network analysis of DEGs associated with ferroptosis

As shown in Fig. 2C, there were 37 ferroptosis-related DEGs filtered into the PPI network complex including 37 nodes and 66 edges based on the STRING database. Average degree of the nodes was 3.57, and the PPI enrichment *P*-value was < 1.0e−16 (Fig. 2C). The top 10 hub genes identified in the PPI network by cytoHubba plugin in Cytoscape software respectively were AKR1C2, AKR1C1, SRXN1, GCLC, TF, TFR2, SLC7A11, CDKN2A, AKR1C3, NQO1 (Fig. 2D).

### Construction of prognostic signatures gene related to ferroptosis in NSCLC

**Fig. 3:**
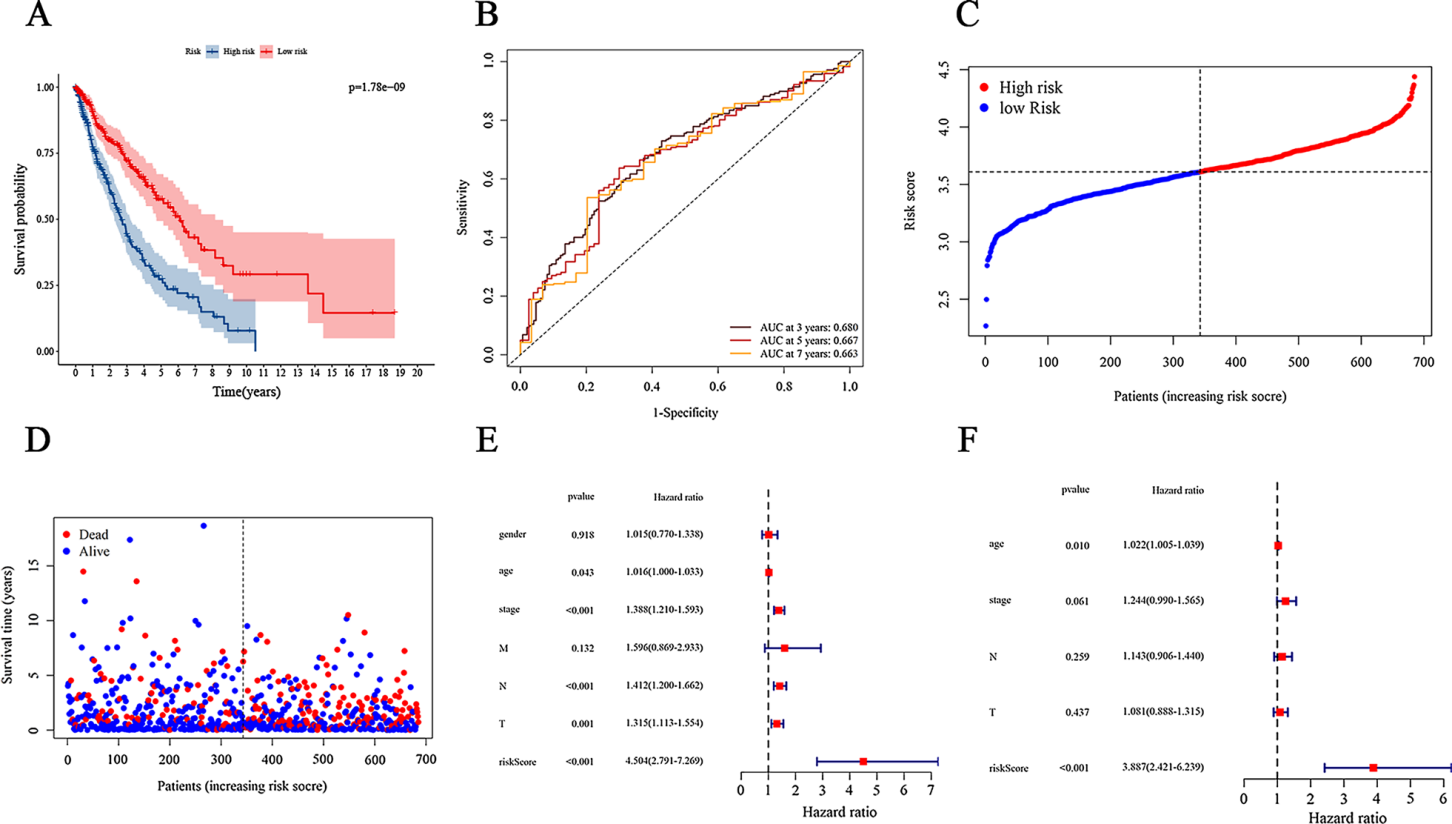
Prognostic analysis of the 6-gene signature model in the TCGA cohort. **A** Kaplan-Meier curves for the OS of patients in the high-risk group and low-risk group. *P* = 1.78e−09. **B** AUC of time-dependent ROC curves in the TCGA cohort. AUC at 3 years = 0.680, AUC at 5 years = 0.667, AUC at 7 years = 0.663. **C** The dotted line indicates the individual inflection point of the risk score curve, by which the patients are categorized into low-risk (green) and high-risk (red) groups. **D** Red dots indicate the dead patients and green dots indicate the alive. With the increase of risk score, more patients died. **E** Univariate Cox regression analysis. Forest plot of associations between risk factors and the survival of NSCLC. **F** Multiple Cox regression analysis. The ferroptosis-related gene signature is an independent predictor of NSCLC

**Fig. 4:**
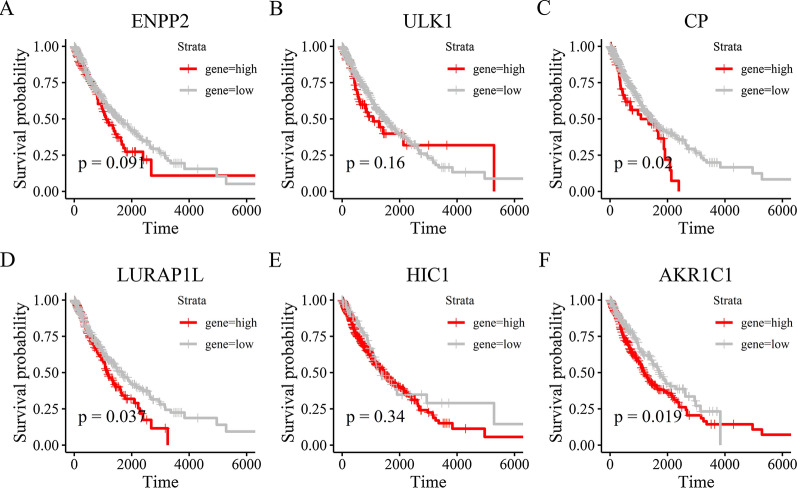
Kaplan–Meier analysis between two risk groups of six genes with prognostic value. **A** ENPP2, *P* = 0.091. **B** ULK1, *P* = 0.16. **C** CP, *P* = 0.02. **D** LURAP1L, *P* = 0.037. **E** HIC1, *P* = 0.34. **F** AKR1C1, *P* = 0.019

Subsequently, we examined the prognostic role of ferroptosis-related genes in NSCLC. Among the NSCLC patients in the TCGA, we identified 37 genes associated with the prognosis of NSCLC through the univariate Cox regression analysis (*P* < 0.05). Next, the multivariate Cox regression analysis identified 6 overall survival associated genes (ENPP2, ULK1, CP, LURAP1L, HIC1, AKR1C1) in NSCLC patients (*P* < 0.05). Additionally, in order to predict prognosis in NSCLC patients, we used six selected genes associated with the NSCLC prognosis to construct a risk model by multiple stepwise Cox regression analysis. Risk score = (0.219 * expression level of ENPP2) + (0.334 * expression level of ULK1) + (0.046 * expression level of CP) + (0.039* expression level of LURAP1L) + (− 0.153 * expression level of HIC1) + (− 0.074 * expression level of AKR1C1). As the survival curve based on TCGA shown in Fig. 3A, samples in low-risk group were associated with a significant increase in survival time compared with the high-risk group (*P* < 0.0001), suggesting higher risk scores predicted worse prognosis. In addition, we also employed receiver operating characteristic (ROC) curves to evaluate the accuracy of the prediction models. And the AUC score was 0.680 (Fig. 3B), indicating a high survival prediction performance of model. As the risk score increases, the patients’ death risk increases, and the survival time decreases (Fig. 3C, D). Besides, univariate and multivariate Cox analyses were combined to analysis the independent predictive factor for NSCLC patients’ prognosis in our model, and it turns out that lasso risk value independently predicted survival expectancy (Fig. 3E, F). Furthermore, Kaplan-Meier analysis was utilized to paint the survival curves and it was compared by the log-rank test based on the threshold of *P* < 0.05 (Fig. 4A–E). Shown in Fig. 4, the high expression of genes CP, LURAP1L and AKR1C1 is strongly associated with poor prognosis in NSCLC (*P* < 0.05), especially the gene AKR1C1 (P = 0.019) whose low expression dictated a prolonged survival time. Thus, we chose gene AKR1C1 for further analysis.

### Immune infiltrates analysis

**Fig. 5:**
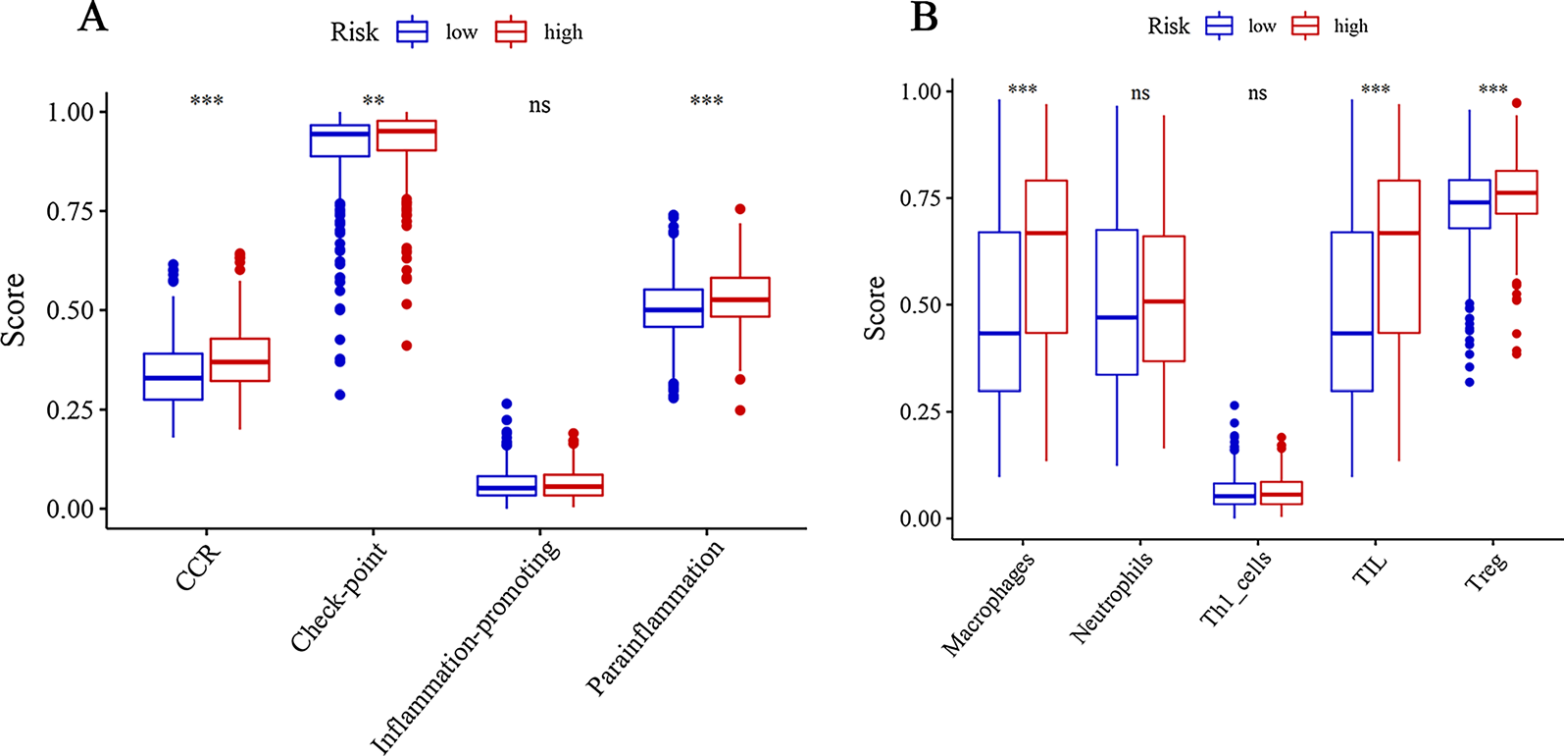
Comparison of the ssGSEA scores between the high-risk and low-risk groups. The scores of 5 immune cells and 4 immune-related functions are displayed in boxplots: **A** Macrophages, Neutrophils, Th1 cells, TIL, Treg. **B** CCR, Check-point, Inflammation-promoting, Parainflammation. Adjusted *P* values were shown as: ns, not significant; *P < 0.05; **P < 0.01; ***P < 0.001

To further explore the relationships between the risk scores and immune cells and related functions, we quantified the enrichment scores of 5 immune cell subpopulations and their related functions with the ssGSEA R package. As the results showed in Fig. 5A, immune cells Macrophages, TIL and Treg in the high-risk group were significantly higher than those in low-risk group. Moreover, the scores of the immune functions, including the CCR, check-point, parainflammation showed the same results, implying their immunological functions associated with ferroptosis were more muted in the low-risk group (Fig. 5B).

### The validation of signature gene

**Fig. 6:**
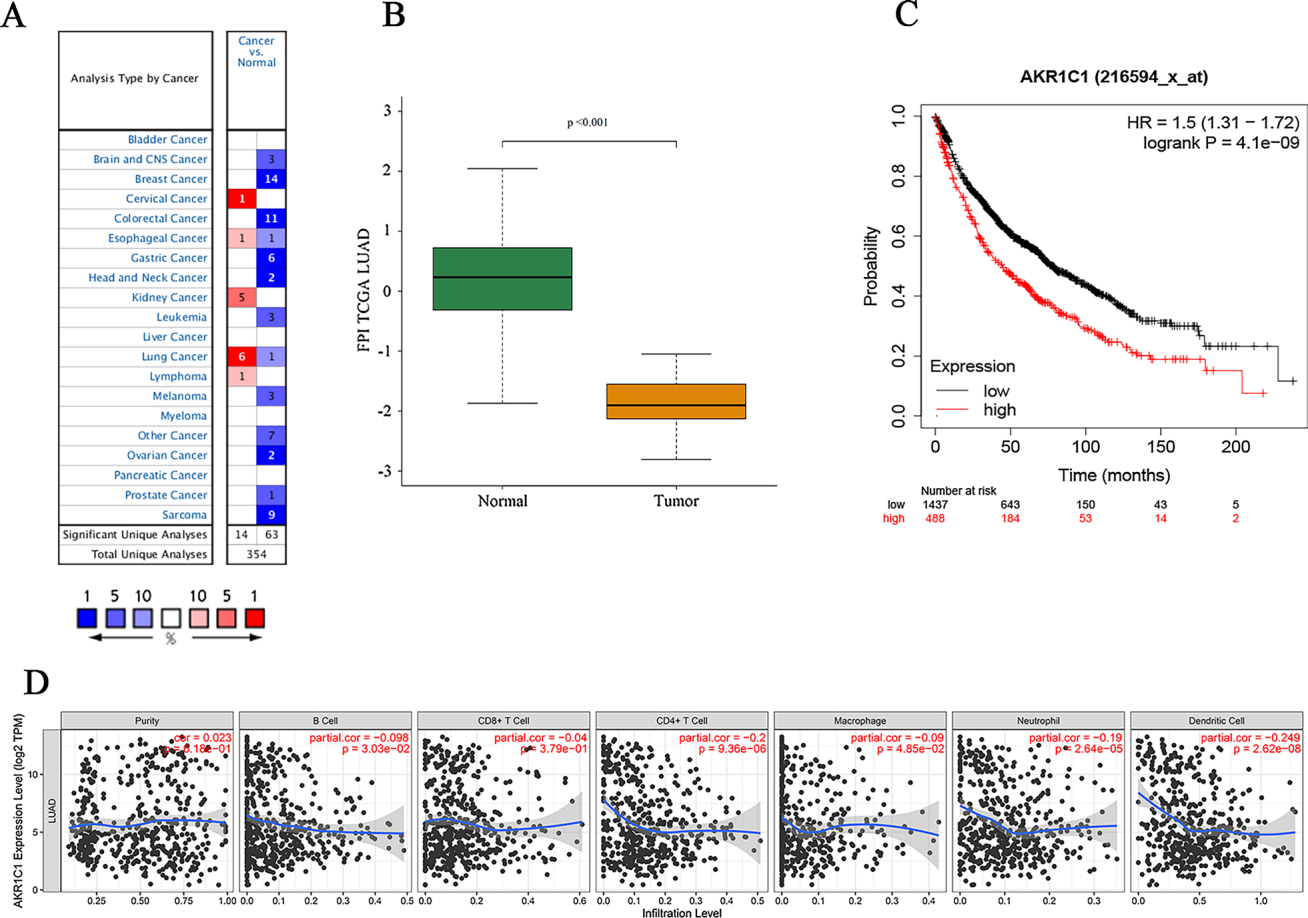
Single gene validation of AKR1C1 in NSCLC. **A** The mRNA expression level of AKR1C1 in various cancer based on Oncomine database. Color images are available online. fold change = 2 and *P*-value = 0.01; **B** The ferroptosis potential index (FPI). AKR1C1 has an FPI < 0.001 in NSCLC. **C** Kaplan-Meier survival curves comparing the high and low expression of AKR1C1 in NSCLC in the Kaplan-Meier plotter database. **D** Correlation between AKR1C1 expression and immune cells infiltration of NSCLC. Tumor purity. AKR1C1 expression was negatively correlated with dendritic cells, CD4 + T cells and neutrophils

**Fig. 7:**
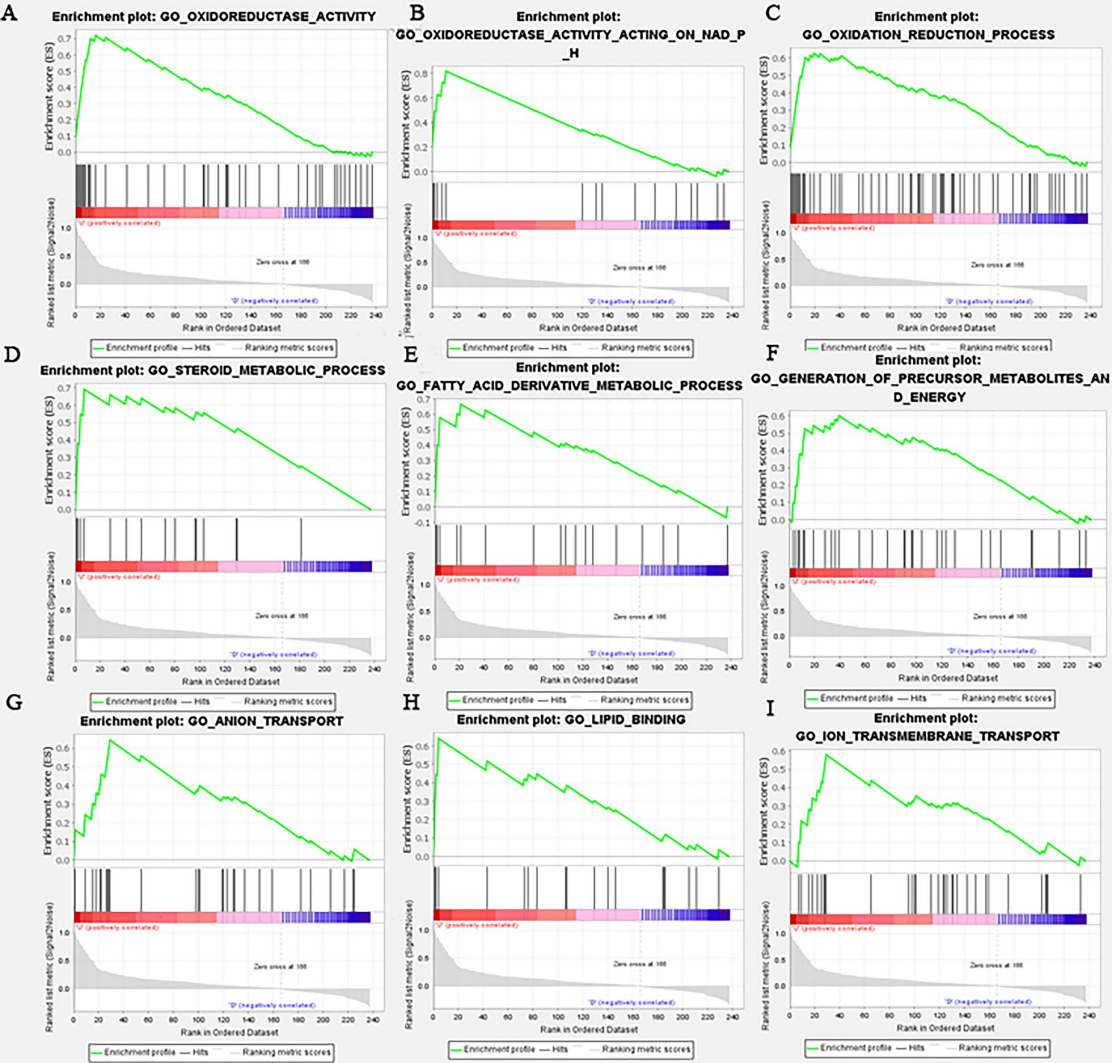
Gene Set Enrichment Analysis (GSEA) validation in NSCLC. **A** xidoreductase activity. **B** Oxidoreductase activity acting on NADPH. **C** Oxidation reduction process. **D** Steroid metabolic process. **E** Fatty acid derivative metabolic process. **F** Generation of precursor metabolites and energy. **G** Anion transport, **H **lipid binding. **I** Ion transmembrane transport

The expression level of AKR1C1 in tumor and corresponding normal tissues in cancer was verified on Oncomine database. As shown in Fig. 6A, AKR1C1 displayed a higher expression level in cervical cancer, esophageal cancer, kidney cancer, lung cancer and lymphoma, especially in lung cancer. Then we checked the impact of AKR1C1 on NSCLC tumor survival rates by the Kaplan-Mayer plotter database (Fig. 6C). The results demonstrated that lower expression level of AKR1C1 was correlated to longer survival time in patients with NSCLC. Besides, in order to further explore the biological function of AKR1C1, we performed GSEA validation on AKR1C1 (Fig. 7A–I). The results in Fig. 7 showed that AKR1C1 were mainly concentrated in biological functions such as biological REDOX, metabolism, metal ions, completing the biological role of AKR1C1 in ferroptosis. Moreover, exploring the ferroptosis level of AKR1C1 in NSCLC, we evaluate the FPI value of AKR1C1 in NSCLC and visualize the results in Fig. 6B which suggested that AKR1C1 was with low ferroptosis level in NSCLC (*P* = 0.001). As shown in Fig. 6D, AKR1C1 mRNA expression level was significantly negatively correlated with infiltrating level of immune cells, including CD4+ T cells (r = − 0.2, *P* = 9.36e−06), neutrophils (r = − 0.19, *P* = 2.64e−05) and dendritic cells (DCs) (r = − 0.249, *P* =2.62e−08). Furthermore, we explored the correlation between the expression of AKR1C1 and the number of 22 types of immune cells infiltrated in NSCLC. According to the results in supplementary Tables 1, we found AKR1C1 expression is associated with the number of various types of immune cells, including B cells naive, B cells memory, plasma cells, T cells CD8, T cells CD4 naive, T cells CD4 memory resting, T cells regulatory (Tregs), T cells gamma delta, NK cells resting, monocytes, dendritic cells resting, dendritic cells activated, mast cells resting and neutrophils (*P* < 0.05, cor > 0.3). Moreover, tumor tissues with higher infiltrating levels of T cells CD8, T cells CD4 naive, T cells CD4 memory resting, T cells regulatory (Tregs), T cells gamma delta, dendritic cells resting and neutrophils had lower level of AKR1C1 expression on cancer cells (Additional file 1: Fig. S1). On the contrary, lower infiltrating levels of B cells naive, B cells memory, plasma cells, NK cells resting, monocytes, dendritic cells resting, and mast cells resting is associated with the high expression level of AKR1C1 in NSCLC. These data suggested the complexity between ferroptosis-related gene AKR1C1 and immunity.

### AKR1C1 silencing inhibits the malignant phenotypes and promotes ferroptosis of NSCLC cells

**Fig. 8:**
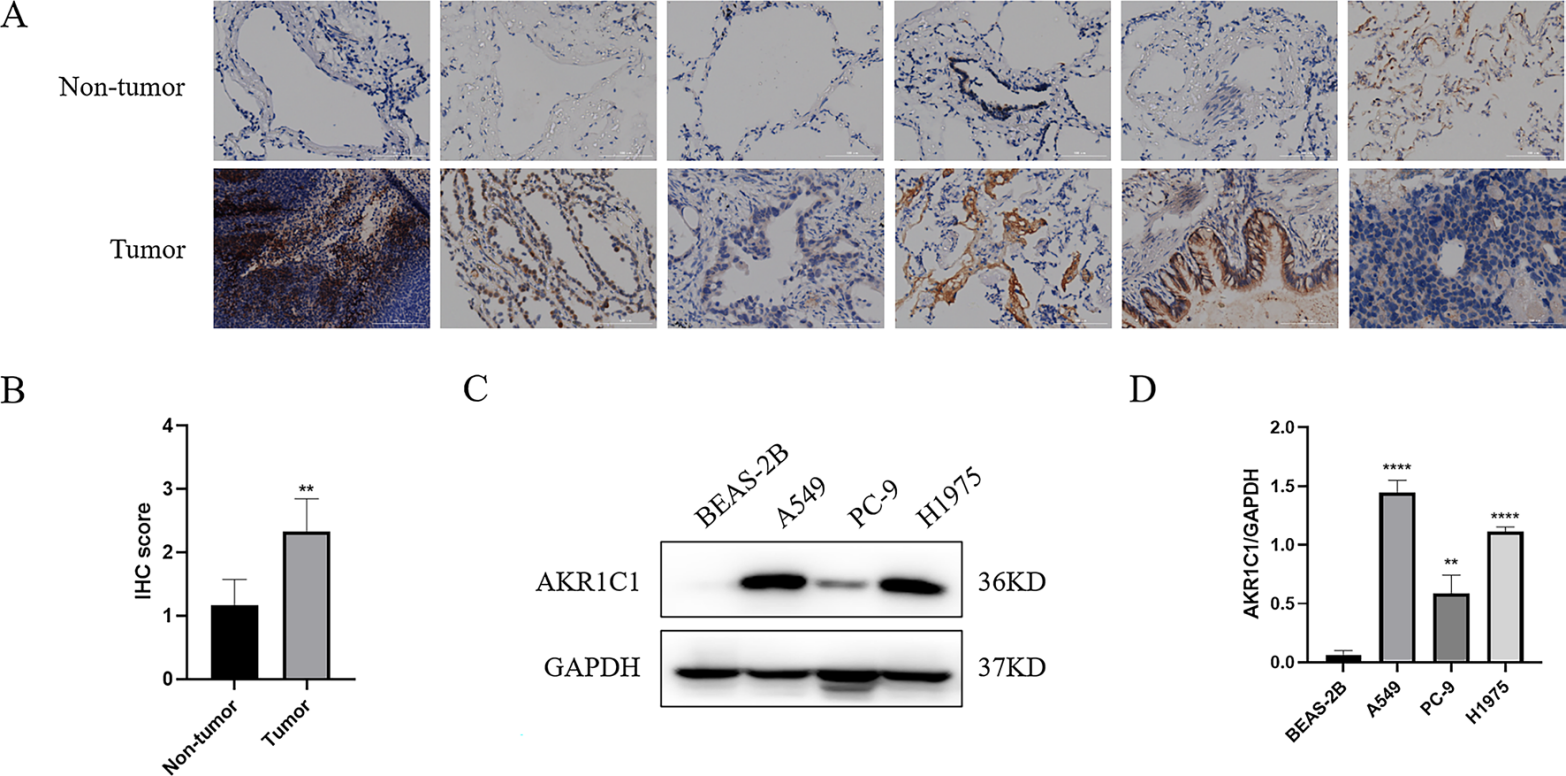
AKR1C1 was upregulated in NSCLC tumor tissues and cell lines. **A**, **B** Representative images and scores of AKR1C1 immunohistochemical staining in six NSCLC and paired non-tumor tissues. **C**, **D** Expression level of AKR1C1 in NSCLC cell lines by western blot. Compared with BEAS-2B, *P < 0.05, **P < 0.01

To determine the clinical relevance of AKR1C1 expression level, we examined AKR1C1 expression in six pairs of clinical NSCLC and corresponding adjacent non-tumor tissue samples. The immunohistochemical assay showed that the expression level of AKR1C1 protein in NSCLC tissues was significantly higher than in corresponding adjacent non-tumor tissues (Fig. 8A, B), which indicated that AKR1C1 was highly expressed in NSCLC. In addition, to further evaluate the expression of AKR1C1 in NSCLC, the protein level was examined in human bronchial epithelial cells and NSCLC cell lines. It was found that the protein level of AKR1C1 was significantly higher in A549, PC-9, and H1975 cells, but almost undetectable in human bronchial epithelial cells BEAS-2B (Fig. 8C, D). Notably, the AKR1C1 level was higher in A549 cells and H1975 cells than in PC-9 cells. Therefore, A549 cells and H1975 cells were adopted for subsequent cell analyses.

**Fig. 9:**
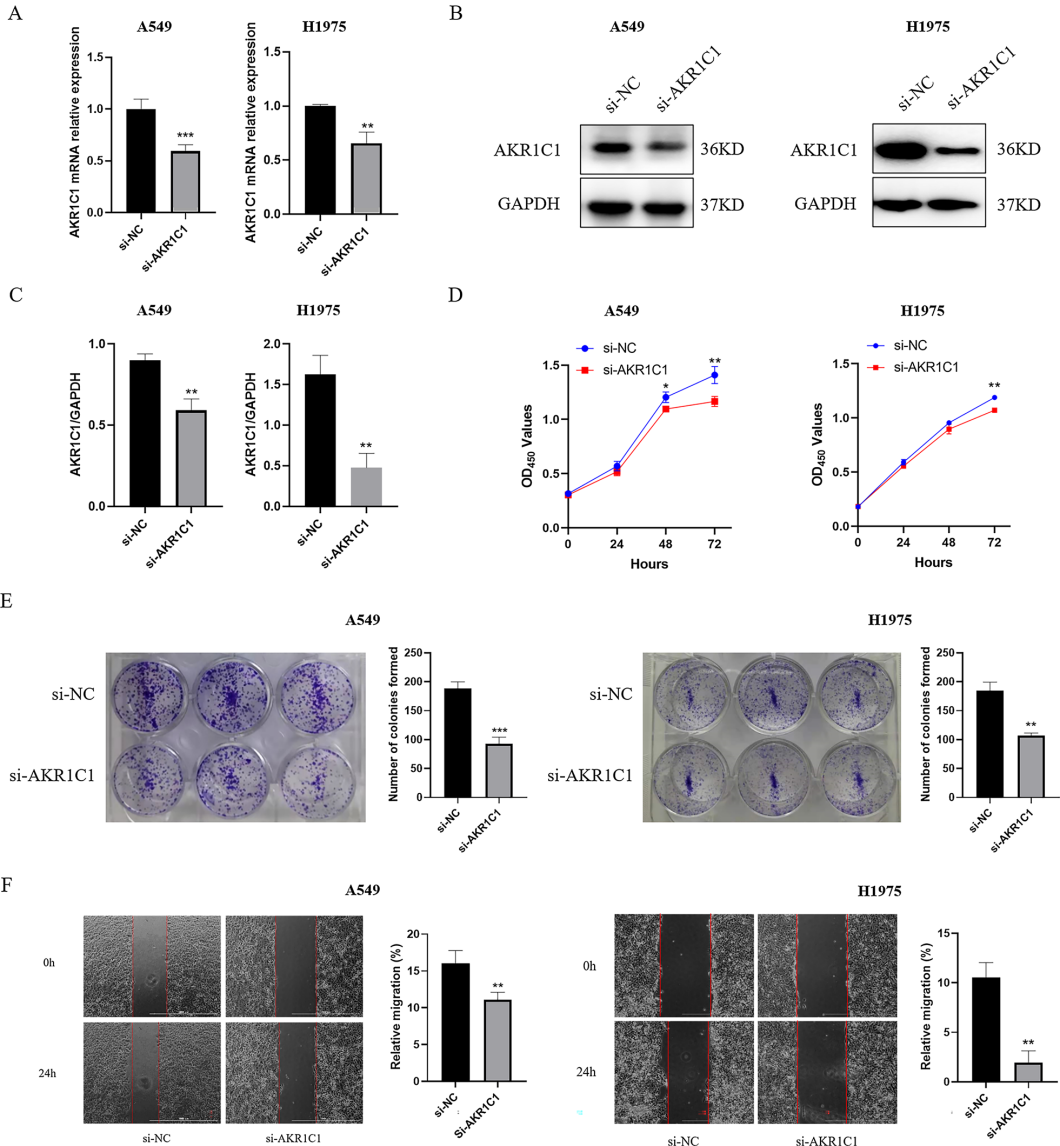
AKR1C1 silencing inhibits the malignant phenotypes of NSCLC cells. **A**–**C** RT-qPCR and western blot results showed that AKR1C1 was successfully knocked out by siRNA in A549 cells and H1975 cells. *represents si-AKR1C1 vs. si-NC, *P < 0.05, **P < 0.01. **D**, **E** The effect of AKR1C1 silencing on cell proliferation were measured by CCK8 assay and colony formation assay. *represents si-AKR1C1 vs. si-NC, *P < 0.05, **P < 0.01. **F** Measurement of wound healing ability. Cells under different treatments were examined for migration to the wound area and photographed

To reveal the biological function of AKR1C1 in NSCLC cells, we transfected A549 cells and H1975 cells using siRNA. RT-qPCR and western blot results showed that AKR1C1 mRNA and protein expression was significantly down-regulated in A549 cells and H1975 cells after si-AKR1C1 transfection, suggesting that AKR1C1 was successfully knocked down (Fig. 9A–C). Subsequently, we performed CCK8 assay and colony formation assay to observe the role of AKR1C1 in A549 cell and H1975 cell proliferation. The results showed that A549 cell and H1975 cell proliferation was significantly decreased in the si-AKR1C1 group compared with the si-NC group (Fig. 9D, E). In addition, the results of wound healing assay showed that the migration ability of A549 cells and H1975 cells in the si-AKR1C1 group was attenuated compared with that in the si-NC group (Fig. 9F). Therefore, our results illustrated that AKR1C1 was essential for the proliferation and migration ability of NSCLC cells.

**Fig. 10:**
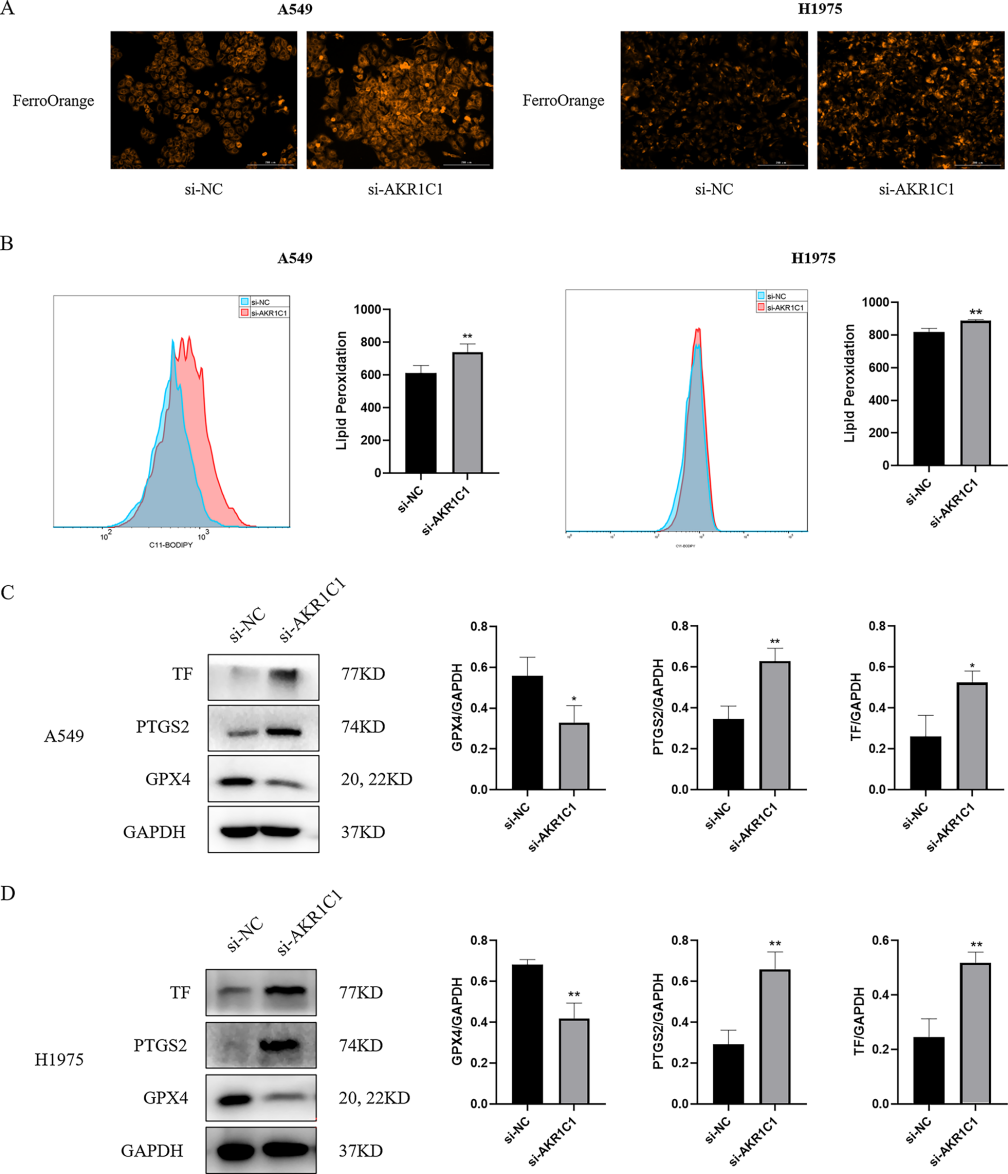
AKR1C1 silencing promotes ferroptosis of NSCLC cells. **A**, **B** Detection of ferrous ions and lipid peroxidation levels after silencing AKR1C1 in A549 cells and H1975 cells. **C**, **D** The expression levels of GPX4, PTGS2, and TF in A549 cells and H1975 cells with AKR1C1 silencing were detected by western blot. *si-AKR1C1 vs. si-NC, *P < 0.05, **P < 0.01

Likewise, to further investigate the role of AKR1C1 expression in ferroptosis, we examined key indicators related to ferroptosis [including ferrous ions (Fe^2+^), lipid peroxidation, GPX4, transferrin (TF) and prostaglandin-endoperoxide synthase 2 (PTGS2)] by interfering with AKR1C1 expression in A549 cells and H1975 cells. First, we examined the effect of AKR1C1 on changes in intracellular Fe^2+^ levels and found that the level of intracellular Fe^2+^ rose in A549 cells and H1975 cells after knockdown of AKR1C1 (Fig. 10A). Moreover, lipid peroxidation plays a key role in the development and progression of ferroptosis. Therefore, we next examined the effect of AKR1C1 on the level of intracellular lipid peroxidation in A549 cells and H1975 cells and showed that inhibition of AKR1C1 expression increased the level of intracellular lipid peroxidation in A549 cells and H1975 cells (Fig. 10B). Meanwhile, the results of western blot showed that GPX4 expression decreased and TF and PTGS2 expression increased in A549 cells and H1975 cells after knockdown of AKR1C1 (Fig. 10C, D). Thus, our results indicated that silencing AKR1C1 promoted ferroptosis in NSCLC cells.

## Discussion

As a newly discovered form of iron-dependent cell death, ferroptosis represents a new perspective for the treatment of cancer and may develop new strategies for the treatment of NSCLC [11, 18]. However, the specific role of ferroptosis in NSCLC has not yet been clarified. In the present study, we found that high expression of the ferroptosis-related gene AKR1C1 was associated with a poor prognosis in NSCLC, and silencing AKR1C1 inhibited proliferation and migration of NSCLC cells and promoted the development of ferroptosis.

Previous studies have demonstrated that human aldo-keto reductase family plays an important role in the metabolism of steroid hormones, metabolism of conjugated steroids, biosynthesis of neurosteroids and bile acids, and synthesis of therapeutic steroids, and is closely associated with NAD(P)(H)-dependent reduction [33, 34]. Aldo-keto reductases can also protect metastatic melanoma from ER stress-dependent ferroptosis [35]. AKR1C1, a member of the aldo-keto reductase family, has been reported to be highly expressed in various types of cancer, such as small cell lung cancer [33], endometrial cancer [36], prostate cancer [37]. It has been shown that high expression of AKR1C1 can promote proliferation and migration of small cell lung cancer cells, and it may represent an independent biomarker for assessing the main prognosis and treatment of small cell lung cancer [33]. Likewise, the results of another study also showed that the loss of AKR1C1 is a good prognostic factor in patients with advanced NPC and increases the chemosensitivity of NPC cells to cisplatin [38]. However, the biological role of AKR1C1 in NSCLC has not been clarified. In the present study, we screened AKR1C1 from the signature genes of a prognostic model and determined that its expression level was significantly upregulated in NSCLC cell lines. Furthermore, to explain the clinical significance of AKR1C1 in NSCLC, we confirmed that high expression of AKR1C1 was strongly associated with poor prognosis in NSCLC patients by Kaplan-Mayer plotter database. And silencing AKR1C1 has been shown to inhibit the proliferation, migration, and increase ferrous ions and lipid peroxidation levels of NSCLC cells. Therefore, AKR1C1 can be used as a specific marker in NSCLC patients.

Tumor progression and the efficacy of immunotherapy are strongly influenced by the composition and abundance of immune cells in the tumor microenvironment [39]. As an important part of tumor microenvironment, tumor-infiltrating immune cells play an important role in NSCLC treatment efficacy and patient prognosis [40–45]. In this study, we confirmed significantly negatively correlation between the expression level of AKR1C1 and the infiltrating levels of CD4+ T cells and dendritic cells on the TIMER database. T cells are an important cellular component of adaptive immunity, and cellular immune responses to prevent tumors are usually attributed to CD8+ T cells [46]. However, increasing evidences have demonstrated that CD4+ T cells play an important role in generating and maintaining anti-tumor immune responses [47–49]. In addition, dendritic cells also play an important role in immune response, and they are the most effective antigen-presenting cells to induce primary immune response in cancer [50]. Current studies have shown that high dendritic cells infiltration in lung cancer is associated with a good prognosis [51, 52], while immunotherapy using dendritic cells has shown good results in clinical trials of lung cancer patients [53, 54]. Considered together, these data suggest that high expression of AKR1C1 might be correlated with immunosuppression in NSCLC.

In summary, the results in this study suggest that AKR1C1 is an important prognostic biomarker that may predict long-term survival in NSCLC patients, which can regulate the proliferation and migration of NSCLC cells and promote the occurrence of ferroptosis. Therefore, AKR1C1 can serve as a potential biomarker of prognostic value in NSCLC and the mechanisms underlying the prognostic value of AKR1C1 in NSCLC deserves further experimental exploration.

## Supplementary Information

**Additional file 1:** Additional table and figure.

## Data Availability

All relevant data and material are within the paper and its Additional files.
