# Supplementary material for: Ferroptosis-related gene AKR1C1 predicts the prognosis of non-small cell lung cancer
Source: Cancer Cell Int. 2021 Oct 26;21:567. doi: 10.1186/s12935-021-02267-2 (PMC8549233; doi:10.1186/s12935-021-02267-2)
Supplement: Supplementary file 1 — Additional file 1: Additional table and figure. [file 12935_2021_2267_MOESM1_ESM.docx]

**Supplementary Data**





Supplementary Figure 1. Correlation between tumor cell AKR1C1 expression and the number of 22 immune cells in human melanoma. two-sided Fisher’s exact test; (A) B cells naive; (B) B cells memory; (C) Plasma cells; (D) T cells CD8; (E) T cells CD4 naive; (F) T cells CD4 memory resting; (G) T cells CD4 memory activated; (H) T cells follicular helper; (I) T cells regulatory Tregs; (J) T cells gamma delta; (K) NK cells resting; (L) NK cells activated; (M) Monocytes; (N) Macrophages M0; (O) Macrophages M1; (P) Macrophages M2; (Q) Dendritic cells resting; (R) Dendritic cells activated; (S) Mast cells resting; (T) Mast cells activated; (U) Eosinophils; (V) Neutrophils.
